# Diachronic analysis of social structure in Italian metropolitan areas: a comparative–historical research and open-access database for urban studies (DIAMETROIT)

**DOI:** 10.3389/fsoc.2026.1764193

**Published:** 2026-05-22

**Authors:** Niccolò Morelli

**Affiliations:** Department of Political and International Studies, University of Genoa, Genoa, Italy

**Keywords:** diachronic studies, functional urban area (FUA), Italian cities, Mediterranean cities, residential segregation by age, Southern European cities, urban grid, urban segregation

## Abstract

Urban research has demonstrated the impact of residential location on life chances and opportunities. This perspective highlights how population structure and territorially embedded opportunities and constraints shape life courses, employment, crime and civic participation. Imbalances in social structure may lead to residential segregation, often with adverse consequences for disadvantaged groups. However, prevailing frameworks—largely developed in rental-dominated or ethnicity-focused contexts—tend to treat dimensions such as age and occupation as analytically independent. DIAMETROIT advances a different view: in home–ownership–centered housing regimes, these dimensions are jointly mediated by the intergenerational transmission of property, which structures access to space and opportunities. In Europe, which is traditionally characterized by strong welfare systems, residential segregation is increasing in major urban areas. This trend calls for a shift in analytical focus from metropolitan cores to broader functional urban areas (FUAs), where economic opportunities and structural constraints increasingly operate. Understanding segregation also requires a diachronic perspective that captures long-term transformations. In Italy, however, comprehensive metropolitan analyses on a fine spatial scale remain limited. This gap reflects both technical and institutional constraints: data aggregation at administrative levels, changing census units and limited longitudinal comparability, alongside chronic underfunding and a fragmented research system. At the same time, the Italian case is theoretically consequential: high home ownership rates and a familistic welfare regime mean that housing access is largely mediated through intergenerational transfers, reinforcing the link between demographic and socio-economic segregation. The DIAMETROIT project focuses on two dimensions: generational and socio-professional segregation. Younger cohorts face increasing precarity in labor and housing markets, yet their spatial distribution remains underexplored. Socio-professional segregation, which is central in European debates, is also gaining relevance in Italy amid stagnant wages and rising costs. Rather than treating these as separate processes, we interpret them as interdependent outcomes of housing structures. By developing harmonized, comparable spatial units and integrating demographic and socio-economic data, the project provides new insights into metropolitan inequalities across Italian FUAs.

## 1 Introduction

The DIAMETROIT project advances a conceptual argument: in Mediterranean home-ownership–centered housing regimes, generational and socio-professional segregation are not analytically independent but are jointly mediated by the intergenerational transmission of property. Prevailing frameworks—developed in rental-dominated or ethnicity-focused contexts—tend to treat these dimensions separately, overlooking their institutional interdependence. By foregrounding housing market structures as the linking mechanism, the Italian case allows us to refine the comparative European framework and specify this relationship empirically.

To situate the contribution of this project, we first discuss the international debate on segregation and its multiscalar nature and then examine how Mediterranean and Italian specificities challenge existing frameworks.

### 1.1 Residential segregation and multiscalarity in international research

Studies on residential segregation occupy a central place in social research, rooted in a strong American tradition and progressively expanding globally (van Ham et al., 2021). Initially focused on ethnic segregation in major U.S. cities (Massey, 2020), this field has evolved to include multiple dimensions of inequality, such as socio-professional differentiation and access to services and infrastructure (Fujita, 2016; Rokem and Vaughan, 2018). This scholarly trajectory draws inspiration from the theoretical framework pioneered by Blau (1960), which conceptualizes how the distribution of individuals across key attributes (age, social class, ethnicity and level of education) shapes their opportunities and constraints, thereby giving rise to vicious cycles of inequality. Among them are segregation patterns. This perspective has been operationalized by Sampson (2012), who, through quantitative and qualitative research, has shown how neighborhood inequalities shape individuals' life paths and influence neighborhood dynamics.

While early research was largely centered on the U.S., similar dynamics have been documented across Europe and beyond (Musterd, 2023). European cities, traditionally characterized by lower segregation levels due to stronger welfare systems, are nonetheless experiencing increasing spatial inequalities along both ethnic and socio-economic lines (Benassi et al., 2023; Tammaru et al., 2015). This trend has prompted a reorientation of research toward the metropolitan scale, as economic opportunities, housing markets, and mobility systems increasingly operate across integrated urban regions rather than within administrative boundaries (Musterd, 2020; Keil et al., 2016). A key development in this literature is the recognition of segregation as a multiscalar phenomenon whose aggregate patterns may diverge significantly from local dynamics. While overall levels of segregation may appear to be stable or declining, processes of concentration often intensify in specific neighborhoods, producing uneven spatial configurations (Clark et al., 2015). This is focusing more attention on the need for spatially sensitive approaches capable of capturing intra-urban variation. At the same time, scholars emphasize the importance of adopting a diachronic perspective, as segregation processes unfold over long periods and reflect cumulative transformations in urban structure (Logan and Parman, 2017; Sabater and Finney, 2023).

Despite these advances, important gaps remain. In particular, the literature has only recently begun to address generational dimensions of segregation, even though age structure significantly affects social organization, service demand and opportunity structures (Sampson, 2012). More broadly, different dimensions of inequality—such as age and socio-professional status—are often analyzed separately, with limited attention paid to the institutional mechanisms that may link them.

### 1.2 Mediterranean and Italian specificities

These limitations become particularly evident in the Mediterranean context, where housing systems and welfare regimes differ markedly from those in which the dominant theories were developed (Daconto and Montesano, 2024). In Italy, residential segregation has not been a central topic of empirical research, particularly regarding socio-professional differentiation, which has remained marginal despite its prominence in other European contexts (Préteceille and Cardoso, 2020; Oberti and Préteceille, 2004). At the same time, generational inequalities—although widely documented in terms of labor market outcomes—have rarely been analyzed in their spatial dimension. This gap reflects both empirical and theoretical factors. The Italian research context has been constrained by limited data comparability over time and the relative fragmentation of research infrastructures. However, it reflects a deeper mismatch between the prevailing analytical frameworks and the institutional configuration of Southern European societies. In Italy, high home-ownership rates, a residual rental sector, and a familistic welfare regime mean that access to housing is largely mediated through intergenerational transfers rather than market allocation. As a result, residential mobility is more constrained, and spatial inequalities are more inertial.

In this context, socio-professional and generational segregation cannot be understood as independent processes. The increasing difficulty younger cohorts face in accessing both stable employment and affordable housing (Luppi et al., 2024) suggests that their spatial distribution is closely tied to the geography of family-owned property. Similarly, socio-professional differentiation is shaped not only by labor market dynamics but also by unequal access to housing resources. This interdependence calls for a reconceptualization of segregation that explicitly incorporates housing systems as mediating structures. Our study builds on this premise by focusing on the Italian case as a critical setting for analyzing the joint dynamics of generational and socio-professional segregation. By adopting a metropolitan and diachronic perspective, it aims to address both the empirical gaps in the literature and the broader need to refine existing theoretical frameworks, considering Southern European specificities.

## 2 Aim and objectives

Before turning to the research questions, it is necessary to make explicit the theoretical commitments that guide the empirical strategy and the specific methodological steps through which they are operationalized. Four commitments structure the analysis. First, following Blau's (1960) study and its contemporary elaboration by Sampson (2012), residential structure is conceived as a configuration of population composition and territorial opportunities that jointly shape life trajectories; this commitment is operationalized through the joint analysis of population structure and the localization of services, economic activities and infrastructure. Second, in line with Musterd (2020), Tammaru et al. (2015) and van Ham et al. (2021), segregation is treated as a multiscalar phenomenon whose global and local expressions may diverge; this commitment is operationalized through the parallel computation of dissimilarity indices across FUAs, cities and municipalities (global), and location quotients (LQs) at the grid level (local), together with Moran's I to assess spatial clustering (Moran, 1950). Third, following Logan and Parman (2017), Salvati (2020), and Sabater and Finney (2023), we treat segregation dynamics as intelligible only over sufficiently long periods; this commitment is operationalized through the diachronic grid harmonization of census tracts across four waves and the inclusion of intermediate decades, not only of the endpoints. Fourth, following the literature on critical junctures (Capoccia and Kelemen, 2007) and its application to European housing markets (Arbaci, 2019; Andersson et al., 2018), we treat the 2008–09 financial crisis as a potential structural break whose effects may differ across tenure regimes. This commitment is operationalized through comparisons of pre- and post-crisis segregation trajectories and through the inclusion of housing market variables (property values and rent levels) in the multivariate models.

Beyond its empirical and infrastructural contributions, this protocol advances a conceptual argument. Frameworks developed in the contexts of mature rental markets or in response to ethnic segregation have treated generational and socio-professional segregations as conceptually independent dimensions of urban inequality (Tammaru et al., 2015; van Ham et al., 2021). The Italian case, analyzed diachronically and at a fine spatial scale, allows us to refine the comparative European framework by identifying a configuration in which housing market mediation bridges generational and socio-professional segregation dynamics. This conceptual specification, rather than the empirical coverage alone, is the distinctive contribution of the study protocol.

## 3 Research questions

The theoretical framework outlined above has been developed largely in contexts where residential allocation is mediated either by a mature rental market (Northern and Western Europe) or by policy instruments addressing ethnic and racial concentrations (the United States). The Italian case is embedded in a distinct institutional configuration whose specificities are not ancillary to the analysis of segregation but constitutive of it. Two institutional features matter. First, the Southern European welfare regime, which is characterized by a familistic, territorial fragmentation of social protection and comparatively limited transfers directed at housing (Ferrera, 1996; Ascoli and Pavolini, 2015; Andreotti and Mingione, 2016), has historically left the family as the primary unit of residential provision. Second, the Mediterranean housing system is dominated by home ownership (Allen et al., 2004; Arbaci, 2019), with a residual rental sector, a negligible social housing stock, and the intergenerational transmission of property as the dominant allocation mechanism (Bernardi and Poggio, 2004; Poggio, 2008). The intersection of these two features has direct implications for segregation analysis. First, segregation dynamics are expected to be inertial, as exit costs from ownership and familial ties constrain residential mobility more strongly than in rental-dominant systems. Second, the residential position of younger cohorts is structurally dependent on the geography of family-owned property, which is itself spatially unequal, meaning that generational segregation cannot be reduced to an income or occupational effect but must be analyzed as a derivative of the patrimonial map of families (Filandri and Bertolini, 2016). Third, socio-professional segregation is refracted through the inherited housing market, rather than directly reflecting labor market dynamics. It follows that, in the Italian case, generational and socio-professional segregation are not independent dimensions but are jointly mediated by housing market structures. This is why the role of housing market functions is best understood analytically as a bridging hypothesis, rather than as an ancillary one.

Building on the theoretical framework outlined above, this study aims to investigate the evolution and spatial configuration of socio-professional and generational segregation in Italian metropolitan areas from 1991 to 2021. In particular, the project seeks to operationalize the relationship between structural opportunities and constraints, residential sorting mechanisms and their diachronic transformation within the specific institutional context of Southern European welfare regimes.

The analysis is guided by the following research questions:

RQ1. To what extent have socio-professional and generational residential segregation levels changed across Italian functional urban areas between 1991 and 2021?
RQ2. How do residential segregation patterns differ across spatial scales (municipality, city and FUA), and to what extent do global and local measures provide divergent interpretations of these trends?
RQ3. What is the role of housing market dynamics, service provision and accessibility in shaping the spatial distribution of socio-professional and generational groups?
RQ4. To what extent has the 2008–09 economic crisis acted as a critical juncture in reshaping residential segregation patterns, particularly through changes in real estate dynamics?

Grounded in the European literature on rising socio-economic inequalities and changing urban dynamics (Tammaru et al., 2016; van Ham et al., 2021), as well as in studies on housing financialisation and generational divides (Sabater and Finney, 2023; De Rosa, 2022), the study advances the following hypotheses:

H1. Multiscalar divergence hypothesis. While overall levels of residential segregation at the metropolitan (FUA) scale are expected to remain relatively stable or even decline, local-level segregation is expected to increase, resulting in more spatially clustered and polarized patterns.
H2. Generational inequality hypothesis. Younger cohorts are expected to be increasingly concentrated in less advantaged and more precarious residential contexts, reflecting growing barriers to housing access and labor market instability, particularly in the post-2008 period.
H3. Socio-professional polarization hypothesis. Socio-professional residential segregation is expected to increase, especially among higher-income and higher-status groups, which are likely to exhibit stronger spatial clustering than lower-income groups.
H4. Changes in housing market conditions—particularly rising property values and rent levels and processes of gentrification—are expected to mediate the relationship between generational and socio-professional segregation. Specifically, housing market dynamics are hypothesized to shape how these two dimensions interact spatially by structuring access to residential locations and reinforcing or attenuating patterns of inequality across groups.
H5. Critical juncture hypothesis. The 2008–09 economic crisis is expected to mark a structural break in residential segregation trajectories, intensifying both generational disparities and the alignment between socio-professional segregation and real estate dynamics.

Among the hypotheses advanced, H4 is specified as a mediating mechanism linking H2 (generational segregation) and H3 (socio-professional segregation), in line with the theoretical framework of Mediterranean housing regimes.

The project will also forecast the creation of an accessible research infrastructure to analyze segregation at the metropolitan level. The creation of this infrastructure will pave the way for researchers nationally and internationally, thereby promoting increased research activities within Italy and between Italy and other countries. This, in the long run, is expected to elevate the scale and scope of urbanization research, making its empirical results a reference point for sociologists, political scientists and planners, particularly for understanding the dynamics of metropolitan neighborhood evolution in the design of future policies.

## 4 Method and analysis

The research project involves a challenging methodological approach that has not been extensively explored thus far. It is essential to note that conducting a diachronic analysis at the sub-municipal level is not impossible; however, it has traditionally been complex and discouraged for research projects with shorter funding durations, unlike those provided by initiatives such as Fondo Italiano per la Scienza (FIS)^1^ or ERC grants.

Moreover, a study of this nature necessitates a multidisciplinary team with deliberate inclusion of geographers, sociologists, statisticians and demographers, who can collaborate closely within a single research center to apply a transdisciplinary strategy to the research objective. This level of collaboration is feasible primarily with funding from initiatives such as the FIS.

One of the primary methodological challenges stems from an operational choice made by the Italian National Institute of Statistics (Istat) and involves not using the same boundaries and numbering for census sections over time. This presents a daunting challenge in data harmonization. However, this challenge can be overcome through team efforts using the grid analysis tool, which has been applied in a European context to compare different cities when the units of analysis are not uniform (Andersson et al., 2018; Benassi et al., 2023; Marcinczak et al., 2023).

The grid analysis methodology involves dividing urban space into uniform grids (e.g., 100 m x 100 m) and associating population, housing and services census data at the census tract level within that space using geolocation applications, thanks to dasymetric techniques. This approach generates fine-grained areas that are harmonized across various census waves, addressing issues related to the non-matching numeric coding of census sections and slight boundary variations. This tool should be applied to population, industry and service census data to better understand the concentration of generational and professional social groups, as well as service and economic activities. Moreover, this methodology can also be used to assign values to other databases and geographies, assigning values to each grid using areal interpolation (De Falco and Irpino, 2024). It is a groundbreaking study that aims to provide, for the first time in Italy and with data internationally comparable, in-depth knowledge of the urban evolution of major metropolitan areas (Rome, Milan, Naples, Turin, Palermo, Genoa, Florence and Bari).^2^ This is the only way to analyze data at the sub-municipal level in Italian cities across census waves, as the Istat changes the shape and the numeration of census tracts at each census (Benassi and De Falco, 2025) but also to reduce the bias induced by comparing territorial units with different shapes and forms, known as the modifiable area unit problem (MAUP; Openshaw, 1984). This approach has already been used in European international empirical research on urban contexts (Alessandrini et al., 2017) and has proven effective in analyzing suburban changes, both for national and international comparisons (Benassi et al., 2023). However, in this project, it will be used for the first time to examine the relationships among population change, the housing market, income dynamics, and service availability. The research project will compare levels of residential segregation across the FUA, including its core and suburbs.

The research aims to explore not only the changes between t0 (1991) and t1 (2021) but also the intermediate decades to provide a more comprehensive understanding of evolutionary processes.

In terms of measurement, we employ established synthetic indices of segregation, most notably the dissimilarity index, computed across multiple spatial scales—FUAs, cities and municipalities—and across all available census waves for the FUAs under study. The dissimilarity index remains one of the most widely used measures in the literature, providing a robust assessment of the evenness dimension of segregation (Massey and Denton, 1988). However, as extensively noted, such global indices do not capture intra-urban spatial variation and are therefore limited in their ability to identify where segregation is occurring within urban systems (Reardon and O'Sullivan, 2004). To address this limitation, we complement global measures with local indicators, particularly the LQ. In the context of residential segregation, the LQ captures the relative concentration of a given population group within a spatial unit compared to its share in a broader reference area (e.g. municipality, city or FUA), thereby identifying patterns of over- or under-representation (Brown and Chung, 2006). The main advantage of this measure lies in its capacity to spatialise segregation processes, enabling the identification of localized clusters and territorial disparities, rather than merely quantifying overall segregation levels. In this sense, the LQ aligns with approaches that emphasize the importance of the local spatial context in segregation analysis (Anselin, 1995; Reardon and O'Sullivan, 2004). In addition, spatial autocorrelation statistics—such as Moran's I—will be employed to assess the extent to which observed patterns of segregation exhibit spatial clustering or dispersion, thus providing further insight into the spatial structure of socio-demographic distributions (Moran, 1950; Anselin, 1995).

Finally, multivariate statistical models will be used to investigate the determinants of generational and socio-professional segregation. In line with previous research, key explanatory variables will include income levels, housing market dynamics (e.g. property values and rents), socio-demographic characteristics, the availability of services and the accessibility indicators to assess their statistical significance in shaping observed segregation patterns (Tammaru et al., 2016; van Ham et al., 2021).

### 4.1 Detailed description of the methodological process

Step 1. Harmonization of Census Section Boundaries across Different Census Waves and Reconstruction of Sub-Municipal Units via Grid Analysis (M1: HARGRID): The initial research process involves harmonizing data between different census waves. This objective will be achieved with the grid analysis tool. This harmonization process aims to address issues related to census section numbering and boundary variations across time. This approach involves dividing urban areas into uniform grids and associating census section data with each grid. It will create harmonized and reliable units of analysis (Month 15).

Step 2. Analysis of Socio-Demographic Structure in the Examined Metropolitan Areas (1991–2021; M2: Metrocompo): In this passage, we will analyze the socio-demographic structure of the selected metropolitan areas, focusing on 1991–2021. Special attention will be paid to generational and socio-professional composition (month 19). The analysis of the social structure from a diachronic perspective represents an original contribution that will be the object of a specific composition (D2).

Step 3. Analysis of the Distribution and Localization of Services, Industry and Public Transport Infrastructure in the Examined Metropolitan Areas (1991–2021; M3: MetroDISIT): We will examine the distribution and localization of services, industry and public transport infrastructure within the selected metropolitan areas, covering 1991–2021. This analysis will help evaluate the presence of structural opportunities and constraints at the territorial level. The results of this analysis, which is carried out in the Italian context, will be the object of a specific publication (D3; month 23).

Step 4. Identification of Socio-Professional and Generational Segregation Levels in a Diachronic Context in the metropolitan core and the suburbs (1991–2021; M4: DiaMetro-SegProGen): This step aims to identify levels of socio-professional and generational segregation from a diachronic perspective, looking at different areas of the FUA: inside the metropolitan core and in the larger FUA, looking at differences and similarities from 1991 to 2021. Dissimilarity indices will first be identified at the municipal, city and FUA levels. Then, the LQ will be calculated. Finally, a regression analysis will be conducted to evaluate the dimensions that contribute to these levels of segregation. The results of this analysis will be the object of specific publications (D4, socio-professional segregation in a diachronic perspective; D5, age segregation in a diachronic perspective; socio-professional and age levels of segregation difference inside the FUA; month 28).

Step 5. Evaluation of the data related to the 2021 new census wave (M5: EVACENSUS). In 2018, Istat launched a new procedure for census operations. In the new procedure, data will come from administrative datasets (e.g. registry offices and employment offices). Only a subset of households will be interviewed to assess the data and the overall process. This new procedure could influence the results of the general census wave and, therefore, our understanding of urban social structure. As this is a new process, we will weigh the latest census data against past waves to estimate the effects of this choice and offer the scientific community possible explanations and interpretations of the results. This evaluation will be beneficial for researchers at universities and at Istat. The results of this analysis will be the object of a specific publication (D7; month 32).

Step 6. Creation of an Open Access Database (M6: METRO-Data Italy): We are building an interactive, accessible platform that provides demographic, socio-economic, and service data for sub-municipal areas, benefiting researchers, planners, policymakers and the general public (D8).

### 4.2 Detailed description of planned activities

Work Package 1. Data Collection and Integration: The research project will commence with an extensive data collection phase. This will include gathering population census data, economic activity data, service and infrastructure census data, and connected maps, focusing on multiple waves of data from 1991 to 2021. These data will be provided by Istat. Data at the sub-municipal level will be collected to enable detailed, granular analyses. Data on the population will include age, sex, nationality, marital status, formal educational attainment and information on working status and commuting. Data from economic activities, services and infrastructure will encompass all types of economic or social activities, from the public to the private sectors, including the presence of institutions, local public and private services, and the localization and distribution of associationism. Information on infrastructure will focus on public transport infrastructure. We will also add diachronic and spatially disaggregated data on housing prices and income.

Work package 2: Geospatial Analysis: The research will incorporate advanced geospatial analysis techniques to map and visualize segregation patterns accurately. Geographic information system (GIS) tools will be utilized to create detailed maps that visually represent census tracts across metropolitan areas from 1991 to 2021.

Work Package 3: Development of Metropolitan Analytical Units. To facilitate diachronic analysis and international comparisons, the research will involve creating analytical units at the metropolitan level using the grid analysis research tool. This will ensure that the findings are directly comparable to similar research conducted in other countries. To improve the technique of areal interpolation, satellite data, such as land cover and built-up volume, will be used (De Falco and Irpino, 2024).

Work Package 4: Statistical Modeling: Advanced statistical modeling techniques, including multivariate regression analysis and spatial statistics, will be utilized to analyze the data. The focus will be on understanding the factors contributing to socio-professional and generational segregation within metropolitan areas and on how these patterns have evolved over the study period.

Work Package 5: Interactive Open Access Platform: A critical aspect of the project is the development of an interactive open access platform. This platform will serve as a user-friendly interface for accessing and visualizing the research results, including segregation data and maps. It will be designed to cater to both the scientific community and the public (D8).

Work Package 6: Publication of Research Findings: Specific publications will be produced based on the research results. These publications will focus on segregation analysis using generational and socio-professional variables, as well as various segregation measures across different analytical levels. A specific publication will be devoted to the study protocol, encouraging other researchers to follow this path.

Work package 6: Dissemination and Collaboration: The research project will actively promote collaboration and knowledge sharing within Italy and internationally. Specific training and dissemination events will be organized to facilitate the creation of international research collaborations. The findings and database will be shared widely to maximize the research's impact.

### 4.3 How to build research teams to obtain these results

The project will involve the recruitment of two researchers, three postdoctoral researchers and two research assistants, structured as follows:

From the project's inception, we will recruit two researchers, three postdoctoral researchers and two research assistants. This core research team will remain engaged throughout the project's duration, offering research assistants the unique opportunity to witness the project from inception to completion and ensuring their seamless integration into the research group. The recruited researchers, in collaboration with the Principal Investigator, will oversee the postdocs and research assistants. During the first year of the project, this research team will focus on analyzing Istat census data covering population, housing, services, industry, and infrastructure. Simultaneously, the team will harmonize this data while closely collaborating with the advisory boards. Such harmonization efforts will involve rectifying the Istat data and spatially harmonizing census sections. These positions are dedicated to analyzing the socio-demographic structure of the examined metropolitan areas, conducting an in-depth study of the localization and distribution of services, industry and infrastructure within these areas, and identifying levels of generational and socio-professional segregation in the metropolitan areas under investigation. They will develop a publicly accessible, interactive research infrastructure for disseminating results. The realization of it will be commissioned to a competent external agency.

In terms of qualifications, researchers will be selected from general and territorial sociology, and postdoctoral researchers will come from demography, social statistics, sociology and territorial sociology. Research assistants will have interdisciplinary backgrounds spanning general and urban sociology, demography, economics and social statistics.

The extensiveness and diversity of this research team are imperative to achieve the ambitious research outcomes detailed earlier. They help establish a multidisciplinary group focused on urban segregation within metropolitan areas, fostering scientific progress for researchers at various career stages. This recruitment strategy not only ensures the efficient execution of this project but also secures a promising future for the research group. The diversity of expertise and backgrounds will be the driving force behind the project's success and the long-term sustainability of the research group.

## 5 Expected results

By the end of the 36-month project timeline, the research aims to achieve multiple significant outcomes that will resonate not only within the scientific community but also across a broader societal spectrum.

At the medium-term horizon, the project aims to provide a comprehensive and diachronic account of socio-professional and generational segregation across metropolitan areas, contributing to a strand of research that has increasingly emphasized the temporal dimension of socio-spatial inequalities (Tammaru et al., 2015; van Ham et al., 2021). We seek to assess the role of generational inequalities in shaping residential choices, with a focus on how these disparities have intensified over time and have been exacerbated by the structural transformations triggered by the 2008–09 economic crisis. A growing body of literature highlights that younger cohorts in Southern European countries, especially in Italy, have faced increasing barriers to housing access, wealth accumulation, and residential mobility due to labor-market precarisation and rising housing costs (Arbaci, 2019; Ascoli and Pavolini, 2015). From this perspective, the crisis can be interpreted as a critical juncture that has amplified intergenerational divides, reinforcing patterns of spatial inequality and residential stratification (Andersson et al., 2018; Checchi et al., 2024).

Despite the centrality of generational inequalities in the Italian scientific debate—particularly in relation to welfare regimes, family structures, and access to home ownership (Naldini and Saraceno, 2022)—their role has remained relatively underexplored within urban and segregation studies, where age is often treated merely as a proxy for other socio-economic variables, rather than as an independent structuring dimension of spatial inequality. However, recent contributions suggest that age and cohort effects deserve greater analytical attention, as they intersect with housing market dynamics and institutional arrangements to produce distinct spatial outcomes (Sabater and Finney, 2023).

In parallel, the project aims to advance the study of socio-professional segregation, a dimension that has received comparatively limited attention in the Italian context despite its prominence in other European research traditions, most notably in France. French urban sociology has long emphasized the spatial differentiation of socio-professional categories as a key axis of urban inequality, documenting strong patterns of occupational segregation within metropolitan regions (Préteceille and Cardoso, 2020; Cousin and Préteceille, 2008). More broadly, comparative European studies have shown that occupational and income-based segregation constitutes a fundamental component of urban socio-spatial restructuring (Musterd et al., 2017; Tammaru et al., 2015).

The relative scarcity of such analyses in Italy is largely attributable to data constraints, particularly the limited availability of fine-grained information on income and socio-professional categories at suburban scales. By leveraging newly harmonized datasets, this project seeks to overcome these limitations and help bridge this gap, aligning the Italian case with the broader European literature on segregation. In doing so, it will provide novel empirical evidence of the interplay between generational and socio-professional dimensions of inequality and their spatial manifestations within metropolitan contexts. By offering internationally comparable, fine-grained metropolitan analysis units, we will lay the foundation for new possibilities in urban research. We will discern the evolving patterns of segregation from 1991 to 2021, creating a pivotal reference for international comparisons. We will ensure a good international impact with dedicated, groundbreaking publications. We will delve into the specifics of generational segregation and socio-professional segregation, as well as various measures of segregation across different analytical levels.

One key objective that will have a positive impact is the creation of an interactive open-access platform. This platform will provide helpful, accessible and innovative data on segregation and its geographical distribution, empowering the scientific community and the public with critical information. It is an infrastructure sorely lacking in Italy, hindering comparative research on metropolitan segregation. With this effort, we aim to elevate Italy to the international stage, making it a hub for comparative studies on metropolitan segregation.

In the long run, the availability of this data infrastructure will catalyze growth in urban research, both in Italy and beyond. It will enable national and international researchers to access a shared research infrastructure that has never been available before.

This collaboration will usher in new possibilities for urban research, positioning us at the forefront of understanding metropolitan dynamics. The project will undoubtedly serve as a point of reference for political scientists, planners and researchers worldwide, offering invaluable insights into the evolution of urban neighborhoods, which are critical for designing future policies and improving the quality of urban life.

Moreover, the emergence of a multidisciplinary research team on metropolitan segregation, supported by this FIS grant, will inspire other researchers to establish large research groups. The DIAMETROIT research team will collaborate to develop valuable expertise that will attract future funding for further exploration and support the research team, ultimately establishing a proper research center for metropolitan structure analysis at the institution.

The expected impact of this project extends far beyond the immediate research community; it has the power to reshape the future of urban studies, urban planning and policy formulation. It will serve as a beacon guiding us toward a more inclusive, data-driven and vibrant urban future. The results it will produce and the research infrastructure it will create will represent the indispensable starting point for future pivotal studies on the effects of pollution and climate change on the population, crime and inequalities in metropolitan areas and for better situating structural opportunities and constraints.

## 6 Ethics and dissemination

The research project does not raise critical ethical concerns. It does not involve the direct participation of human subjects, as it relies on pre-existing, anonymised and aggregated data provided by Istat, which has been approved by the Italian Data Protection Authority (Garante della Privacy). Furthermore, the principal investigator has received comprehensive training in privacy and personal data protection. The host institution also maintains an ethical committee dedicated to overseeing the ethical aspects of all ongoing research projects within the university.

## 7 Limitations

Even if a large amount of data harmonization is done, there are elements this project will not be able to manage. First, the information collected in each census wave can differ, which will affect the harmonization of variables. For example, the socio-professional categories have been explicated differently across census waves. This problem obliges us to go only slightly into the socio-professional analysis, relying solely on the first level of aggregation of the International Standard Classification of Occupations (ISCO-88). This will limit the depth of our analysis, but at this point, it will enrich the socio-spatial analysis of residential segregation by socio-professional groups.

Another limitation is the availability of data from the 2021 census. As mentioned earlier, in 2018, Istat launched the permanent census, with a new methodology that is currently under experimentation. This methodology relies on databases that provide updated population data each year, rather than every 10 years. However, for the census, these databases do not cover all the variables and dimensions previously monitored by Istat. Especially at the suburban level, the risk of receiving unsatisfactory information remains a major concern for scholars. Moreover, information is not organized in a timely manner. For example, for 2026, data related to housing conditions and home property at the suburban level are not yet accessible.

## Footnotes

1 FIS stands for Fondo Italiano per la Scienza. Is the new opportunity launched by the Italian Ministry of University and Research to fund ambitious research projects. The structure of this call is similar to the ERC funds. This project has been funded under the FIS (edition) 2, Starting Grant scheme for 1.3 million euros in the SH3 panel.

2 The choice of cities has followed the criteria of number of residents and geographical homogeneity. Other cities could be included, but as the harmonization process will be particularly time consuming, a choice had to be made.
